# Cellular Biology of Tau Diversity and Pathogenic Conformers

**DOI:** 10.3389/fneur.2020.590199

**Published:** 2020-11-12

**Authors:** Sang-Gyun Kang, Ghazaleh Eskandari-Sedighi, Lenka Hromadkova, Jiri G. Safar, David Westaway

**Affiliations:** ^1^Center for Prions and Protein Folding Diseases, University of Alberta, Edmonton, AB, Canada; ^2^Department of Biochemistry, University of Alberta, Edmonton, AB, Canada; ^3^Department of Neurology and Pathology, Case Western Reserve University, Cleveland, OH, United States

**Keywords:** cell-to-cell transmission, liquid-liquid phase separation, molecular heterogeneity, tauopathy, transgenic mice, ubiquitin-proteasome system

## Abstract

Tau accumulation is a prominent feature in a variety of neurodegenerative disorders and remarkable effort has been expended working out the biochemistry and cell biology of this cytoplasmic protein. Tau's wayward properties may derive from germline mutations in the case of frontotemporal lobar degeneration (FTLD-MAPT) but may also be prompted by less understood cues—perhaps environmental or from molecular damage as a consequence of chronological aging—in the case of idiopathic tauopathies. Tau properties are undoubtedly affected by its covalent structure and in this respect tau protein is not only subject to changes in length produced by alternative splicing and endoproteolysis, but different types of posttranslational modifications that affect different amino acid residues. Another layer of complexity concerns alternate conformations—“conformers”—of the same covalent structures; *in vivo* conformers can encompass soluble oligomeric species, ramified fibrillar structures evident by light and electron microscopy and other forms of the protein that have undergone liquid-liquid phase separation to make demixed liquid droplets. Biological concepts based upon conformers have been charted previously for templated replication mechanisms for prion proteins built of the PrP polypeptide; these are now providing useful explanations to feature tau pathobiology, including how this protein accumulates within cells and how it can exhibit predictable patterns of spread across different neuroanatomical regions of an affected brain. In sum, the documented, intrinsic heterogeneity of tau forms and conformers now begins to speak to a fundamental basis for diversity in clinical presentation of tauopathy sub-types. In terms of interventions, emphasis upon subclinical events may be worthwhile, noting that irrevocable cell loss and ramified protein assemblies feature at end-stage tauopathy, whereas earlier events may offer better opportunities for diverting pathogenic processes. Nonetheless, the complexity of tau sub-types, which may be present even within intermediate disease stages, likely mitigates against one-size-fits-all therapeutic strategies and may require a suite of interventions. We consider the extent to which animal models of tauopathy can be reasonably enrolled in the campaign to produce such interventions and to slow the otherwise inexorable march of disease progression.

## Introduction

Propelled by documentation of tau accumulation in a variety of neurodegenerative disorders and a causal role in some instances, as defined by *MAPT* mutation kindreds, the past two decades of work has seen both remarkable efforts applied to this errant cytoplasmic protein and new insights into its biology and pathobiology. Some strides in understanding have been helped by the availability of corresponding rodent models, but advances in this period have also arisen from the emergence of new, generalized biological techniques such as inducible pluripotent stem cells, three-dimensional cell cultures, optogenetics, gene editing, and cryo electron microscopy (cryo-EM)—to name but a few. In the conceptual realm, prion replication mechanisms of templated protein misfolding derived from study of the prion protein (PrP) have been instrumental in considering how tau disease events are perpetuated inside cells and also how they might spread in an infection-like manner between cells. Similarly, the new understanding that there are forms of protein folding and assembly unrecognized by earlier textbook concepts of secondary, tertiary and quaternary structure is also gaining influence. Thus, the concept of liquid-liquid phase separation (LLPS) of proteins arising from curiosity-driven insights into the why's and wherefore's of low complexity domains in proteins has been extended to encompass DNA-binding protein 43 (TDP-43), heterogeneous nuclear ribonucleoprotein A1 (hnRNPA1) and fused in sarcoma (FUS) in neurodegenerative diseases and now tau itself. Here we recap the remarkable diversity of covalent and conformational variants of tau in tauopathies and some parallels in diverse cell biological effects, these including transit within cells of the same lineage type and across cell lineages. We present an emphasis upon subclinical events, noting that irrevocable cell loss and ramified protein assemblies feature at end-stage tauopathy, whereas earlier malleable events may offer better targets for diverting disease processes. We also discuss uses and limitations of animal models of tauopathy to produce interventions and the trend toward use of low-expresser rodent transgenic models as slower, yet potentially more accurate, embodiments of disease pathogenesis.

In terms of the disease landscape to be considered here, there are no <27 tauopathies described to date. Some examples include frontotemporal lobar degeneration associated with *MAPT* mutations (FTLD-MAPT), Pick's disease (PiD), corticobasal degeneration (CBD), progressive supranuclear palsy (PSP), globular glial tauopathy (GGT) and argyrophilic grain disease (AGD) (1, 2), with considerations of effects in different cell lineages included in Section cell lineages harboring abnormal forms of tau. All tauopathies share the common feature of tau aggregation and deposition in the brain. They are also categorized into two subgroups: primary and secondary. Primary tauopathies are the diseases in which tau aggregation plays a prominent role in disease pathogenesis. In secondary tauopathies, the disease is fueled by defects of other proteins or by tissue trauma which then lead (by means that are sometimes debated) to changes in the repertoire of tau molecular species. Alzheimer's disease (AD) and the inherited prion disease Gerstmann–Sträussler–Scheinker syndrome (GSS) may be classified as secondary tauopathies (3). Arguably the most well-studied tau accumulations are paired helical filaments (PHFs), the principal constituent of the neurofibrillary tangles (NFTs) in AD patients. These filaments consist of two structurally distinct parts: an external “fuzzy coat” comprised of N- and C-terminal areas of the protein that can be removed by treatment with the broad-spectrum protease pronase and then a distinct pronase-resistant region, closer to the center of the protein, containing the tandem microtubule-binding repeats (4); the domain structure of tau will be considered in more detail in Section tau physiology, spliced forms and posttranslational covalent variations.

**Figure 1:**
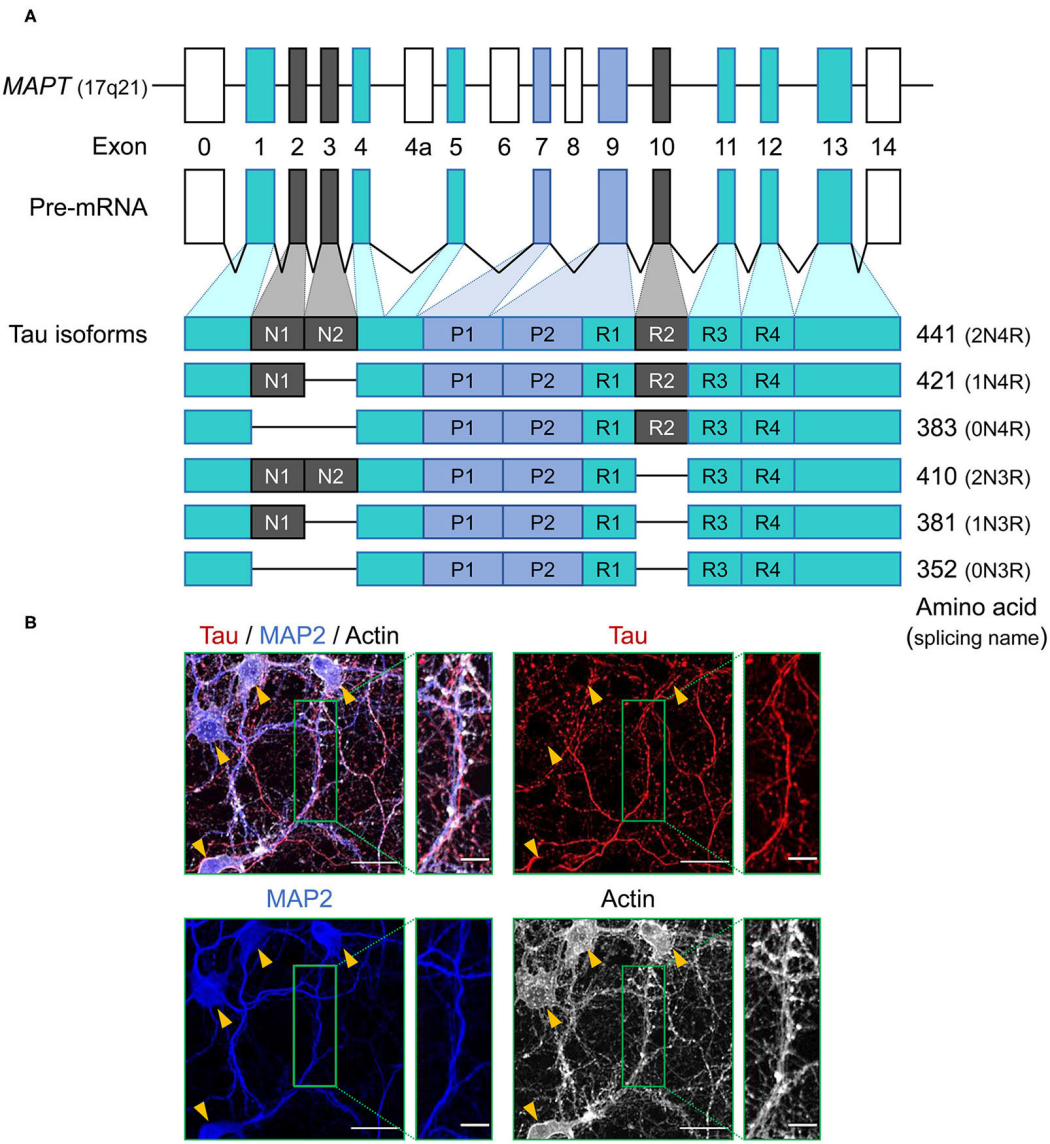
Schematic representation of *MAPT* and the splice isoforms of tau in the human brain. **(A)** Human *MAPT* contains 16 exons. Exons in turquoise boxes (exons 1, 4, 5, 7, 9, 11, 12, 13) are constitutive, while the others are subject to alternative splicing. Exons 0 and 1 encode the 5′ untranslated sequences, and exon 14 is part of the 3′ untranslated region. Exon 4a, 6, and 8 are transcribed only in peripheral tissue, and alternative splicing of exon 2, 3, and 10 generates the six isoforms of tau. Tau isoforms translated from mRNAs that include exon 10, which encodes an additional microtubule-binding motif, are commonly referred to as four-repeat (4R) tau isoforms, whereas isoforms that exclude exon 10 are referred to as three-repeat (3R) tau isoforms. **(B)** Immunocytochemistry of tau (red) in primary hippocampal neurons at 21 days *in vitro* culture using anti-tau monoclonal antibody (RTM47 detecting 2-44 amino acid) with microtubule-associated protein 2 (MAP2) counter stains in blue and actin stain with TRITC-labeled phalloidin in gray. Yellow arrowheads indicate neuronal soma. Scale bars, 30 μm and 10 μm in the boxed images. Image: L. Hromadkova.

FTLD-MAPT [see (5)], as per its name, revolves around *MAPT* mutations and is a neuropathological correlate of frontotemporal dementia (FTD). With respect to the goal of explaining tau diversity in chemical and cell biological terms, as FTLD-MAPT is a primary tauopathy caused by germline mutations, it seems potentially easier to understand the pathogenic process than in cases of idiopathic (sporadic) forms of AD and FTD that lack such mutations. In short, it is perhaps an advantageous model for delineating steps in disease progression. Even so, FTLD-MAPT pathogenesis is not automatically straightforward, noting that cases harboring the same mutation can have a diversity of clinical phenotypes (6–8) including different neuropathological findings. Perhaps surprisingly given a transcriptional profile defining tau mRNA mainly in neurons, unusual forms of tau protein can be found in astrocytes and oligodendrocytes (see below). This is generally the case for FTLD-MAPT with different types of tau mutations and the situation holds for the specific case of the P301L mutation, a case which we have studied in detail using biopsy material from an Iberian P301L kindred with a founder effect mutation (9, 10). Due to the position of the P301L mutation in exon 10 encoding microtubule binding repeat 2, it only affects the 4R form of tau (Figure 1A); in this respect, it is notable that P301L pathologies in astrocytes and oligodendrocytes resemble other 4R-tauopathies such as CBD (11). In short, a recurring observation for the FTLD-MAPT pathogenesis is phenotypic heterogeneity. To begin to grapple with this diversity, we will first consider some cardinal features of tau biochemistry and cell biology.

## Tau Physiology, Spliced Forms, and Posttranslational Covalent Variations

Tau protein is a microtubule associated protein (MAP), encoded by a single gene, *MAPT*, located on chromosome 17q21 of the human genome and consisting of a total of 16 exons (12) (Figure 1A). Tau mRNAs are mainly expressed in neurons and exhibit a developmental change in the ratios of spliced forms (13). Tau mRNAs and proteins have also been detected in oligodendrocytes and astrocytes, but often to a lesser extent (14–20). This observation presents an interesting twist when considering the accumulation of aberrant and potentially pathogenic tau protein in glial cell populations (as considered further below). Primary transcripts of *MAPT* undergo alternative splicing events which, upon translation, yield six protein isoforms in the adult human brain. These spliced mRNA forms differ from each other by the presence or absence of exons 2, 3, and 10. Tau mRNAs that include exon 10, which encodes an additional microtubule-binding repeat (repeat 2), are commonly referred to as four-repeat (4R) tau spliced forms while mRNAs that exclude exon 10 are referred to as three-repeat (3R) tau spliced forms (Figure 1A). The distribution of spliced isoforms is inter-species variable, affected by brain development stage and varies in both temporal and spatial patterns on cellular and brain regional levels (21–25). *MAPT* knock-out mice develop normally without displaying any overt histological abnormalities, possibly due to tau function being rescued by other MAPs, but morphological phenotypes include a minor decrease in microtubule-stability of small caliber axons and some effects on axonogenesis (26, 27). Translation of these mRNAs yield a protein product distributed predominantly in the neuronal axons, but also found in various cellular locations.

Seen in broad overview, tau is a soluble hydrophilic protein described primarily as an essential factor for microtubule assembly (28). The more acidic N-terminal region is mostly involved in interactions with numerous tau binding partners (even affecting its association with cell and nuclear membranes, etc.) (29) while the positively charged C-terminal region encompasses three or four imperfect repeat domains and plays a crucial role in tau interactions with microtubule proteins (although this region can also be involved in interactions with other proteins) (30). Superimposed on this sketched ground-plan, alternative splicing of exons 2, 3 to make mRNAs encoding 0N, 1N, and 2N proteins isoforms can affect the natively disordered N-terminal region in respect to its binding properties with tau-interacting partners and, thus, even the cellular distribution of the protein (23). The N-terminal can itself be perceived as having sub-regions including an acidic region subject to alternative splices and, most notably, a proline-rich domain that can accept many phosphorylation events. The C-terminal region is home to tandem microtubule-binding repeats and subject to the 3R vs. 4R mRNA splicing already noted. These microtubule-binding repeats are followed by a C-terminal extension. Tau lacks any putative signal peptides, transmembrane helices, lipidation or glycolipidation sites that might integrate it into cell membranes and, while it is reported to have short amphipathic helices that might allow it to interact with membrane lipids (31), it is typically considered to be a “cytoplasmic” protein, albeit one that can end up in different cytoplasmic niches or compartments that abut the cytoplasm.

Despite an incomplete understanding of the functional implications of tau distribution among various cellular compartments, tau may be inferred to be multifunctional (32). The most well-studied function is the ability to regulate microtubule assembly and axonal transport of vesicles and organelles (33, 34). Unlike MAP2 which is another major species of MAPs found in the neuronal bodies and dendrites, tau is abundant in the axon (35) (Figure 1B). Tau localization to the other types of microtubule architectures such as growth cones (36, 37) and mitotic spindles (38) are indicative of its dynamic nature and functional repertoire extending beyond axonal microtubule polymerization to encompass developing or regenerating neurites and cell division processes. In neurons, tau has been identified in the synapses (39, 40) and might be involved in the regulation of morphological plasticity of dendrites (41, 42). Provocatively, tau can be released during neuronal activity, an effect which is inferred to involve presynaptic events (43, 44). Tau also binds to and protects neuronal DNA under stress conditions (e.g., oxidative and mild heat stresses) (45, 46) and participates in RNA metabolism through direct association with RNA-binding proteins (47, 48).

**Figure 2:**
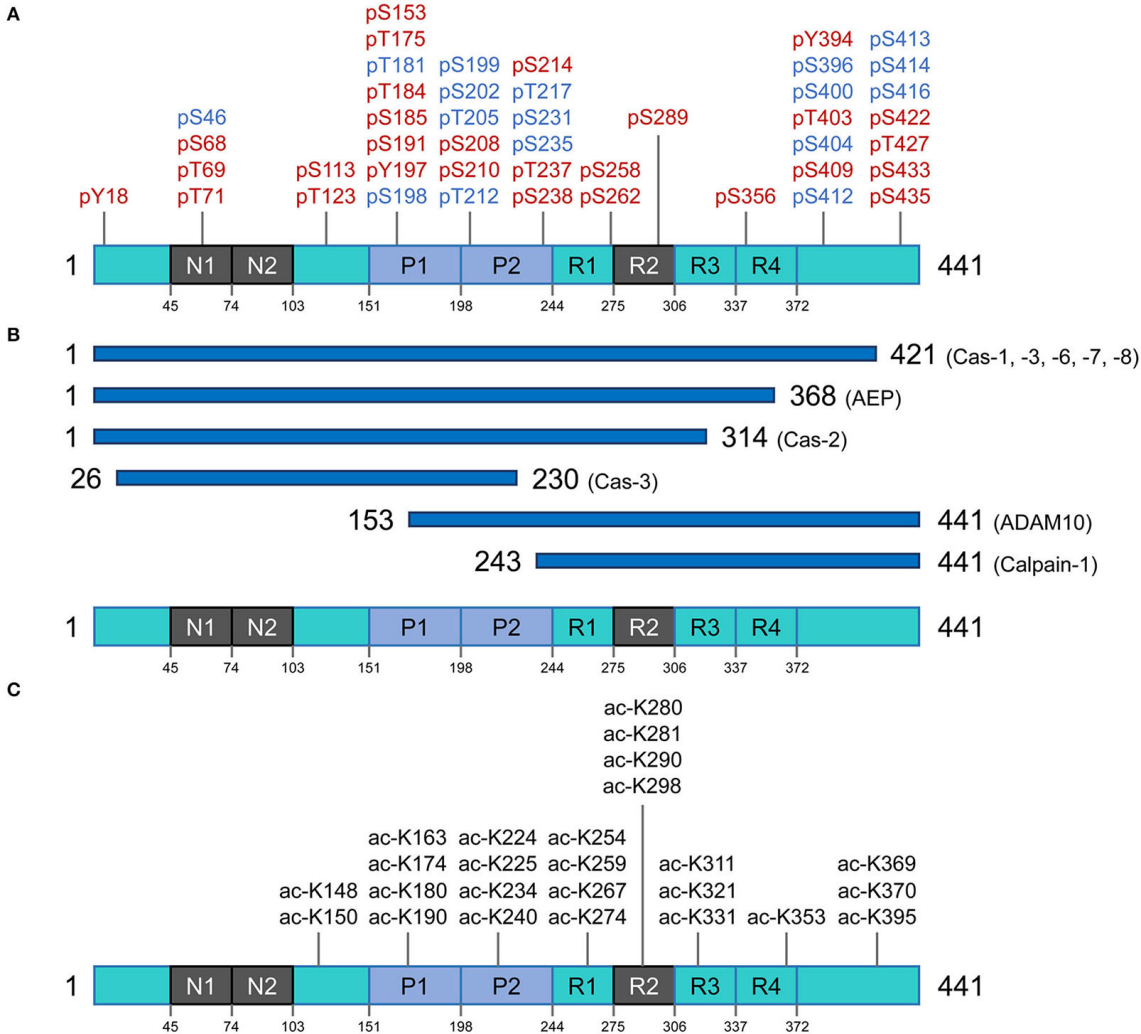
Post translational modifications of the tau protein. **(A)** Phosphorylation residues. Phosphorylated residues found in pathological conditions are represented in red, while phosphorylated residues in blue are observed in both normal and diseased conditions. **(B)** Proteolysis of tau. Potential pathological signatures of tau fragments and the proteases in charge of the cleavage are presented. Further details are described in Table 1. **(C)** Acetylation residues. Acetylated Lys residues in pathological conditions are represented on the longest isoform of human tau (2N4R). This specific modification mainly targets the residues in the core region (Pro-rich domain and repeat domains). Cas, caspase; AEP, asparagine endopeptidase; ADAM10, disintegrin and metalloproteinase domain-containing protein 10.

Descriptions of biochemistry and cell biology need to consider tau species as they behave physiologically vs. unequivocal disease-associated forms of tau (and there are also versions that might fall into to a middle ground). These issues are apparent from the association of different diseases with 3R- and 4R-tau and continue when one turns to another form of covalent variation, namely post-translational modification (PTM). Tau PTMs are striking and important and we have attempted to touch upon physiological and pathological versions of these. PTM's are described in overview in Figure 2A and are inventoried as follows:

### Phosphorylation

Tau phosphorylation is arguably one of the most well-known and abundant PTMs targeting this protein. With 85 potential phosphorylation residues in the longest human isoform (441 amino acids) (45 Ser, 35 Thr, and 5 Tyr residues) (56), this protein is a notable target for several kinases and phosphatases. In consequence, tau's phosphorylation state represents the sum total of dynamic processes (57) and, in turn, regulates different capabilities of tau such as its interaction with the microtubule network and assembly, modulation of cell polarity, axonogenesis, and subcellular localization. Under-phosphorylated tau with phosphorylation of ~1–3 residues is an efficient microtubule network stabilizer whereas hyperphosphorylated tau can have less interaction with microtubule proteins and hence can be more prone to misfolding and consequent aggregation (58). Noting the caveat that some phosphorylation sites are believed to have protective effects and inhibit tau from aggregation and formation of toxic species (59), a broad perspective is that imbalances between tau kinase and phosphatase activities may trigger the non-physiological tau phosphorylation with all the consequences leading to neurodegeneration (60, 61).

A higher amount of phosphorylation in AD brains vs. control brains is extensively documented; normal brain tau has 2–3 mole of phosphate per mole of protein, but AD brains contain tau with an ~3-fold greater stoichiometry (62). So far, 45 phosphorylation sites were detected in insoluble aggregates of tau extracted from AD brain, herein referred to as PHF-tau, several of them being strictly AD-specific and some being shared with tau preparations isolated from control brains (57, 63). Moreover, some clusters of phosphorylation (e.g., Ser210-Thr217, Thr231-Ser238) are involved in a hierarchy of events, meaning that phosphorylation occurs sequentially with initial phosphorylation sites priming subsequent phosphorylation events on nearby residues (64, 65). The phosphorylation sites in PHF-tau are predominantly located in the proline-rich domain and the regions flanking the microtubule-binding domain (63, 65–68), and are involved in alterations in tau microtubule binding dynamics and interactions with other reactive partners. Four phosphorylated residues occurring specifically in PHF-tau have been identified in the microtubule-binding domain region (Ser258, Ser262, Ser289, and Ser356) and were shown to have an impact on microtubule binding capacity (69, 70). Some of the proposed mechanisms of toxicity for hyperphosphorylated tau species include: mis-sorting from axons to the somato-dendritic compartment, disruption of intracellular proteostasis network, interference with nuclear-cytoplasmic transport and dysregulation of physiological functions by altering the repertoire of protein interactors (71–74).

### Proteolysis

As a result of proteolytic processing, generated fragments could behave differently compared to the full-length protein regarding (i) conformation, (ii) solubility, (iii) stability and half-life, (iv) cell localization, and (v) interacting molecules. Under normal circumstances, when tau is no longer needed, it can be efficiently targeted for cytoplasmic degradation pathways such as the ubiquitin-proteasome system (UPS) and autophagy-lysosome system as an attempt to maintain proteostasis (75). However, in disease conditions, tau can become the target of several endoproteases and produce fragments of different sizes that can be found within intracellular tau inclusions and can also be detected in cerebrospinal fluid (CSF), interstitial fluid (ISF) and plasma of patients with different tauopathies. Proteolytic fragments of tau have been shown to be secreted in a variety of cell systems and animal models [reviewed in (76)]. Generation of some of these fragments has been correlated with accumulation of pathologic tau entities and disease progression in several animal models, as well as in post-mortem tissue extracted from tauopathy patients. Moreover, the process of fragmentation is likely superimposed on the phenomenon of tau spreading (below) to generate species with more or less spreading capability. Figure 2B and Table 1 summarize some of these well-characterized cleavage events, the cognate protease and potential relevance to disease conditions. The list of identified tau fragments is longer though, entailing several orphan fragments with corresponding proteases yet to be identified [reviewed in (77)].

**Table 1:** Proteolysis events and fragmentation of tau.

**Cleavage site**	**Identified fragment**	**Protease**	**Impacts of cleavage on disease pathogenesis**	**References**
D421-S422	1–421 (Tau-C)	Cas-1, −3, −6, −7, and −8	Faster aggregation rate compared to full-length tau. Found associated with NFTs in AD brains and increased in AD, FTD-tau, and PSP compared to control brain samples.	(49, 50)
D314-L315, D421-S422 (*in vitro*)	1–314 (tau314)	Cas-2	Lower propensity to aggregate compared to tau441. Impaired synaptic transmission and drives hippocampal neuronal loss.	(51)
D25-Q26, R230-T231	26–230 (20–22 kDa)	Cas-3	Exacerbating mitochondrial dysfunction. Caused NMDAR-mediated cell death in rat CGCs.	(52)
N368-K369	1–368	Asparagine endopeptidase	Reduced ability to induce microtubule polymerization, triggered apoptosis. The C-terminal fragment has increased propensity to aggregate into PHFs compared to tau441.	(53)
A152-T153	153–441 (Tau-A)	ADAM10	Found in serum from patients with AD and inversely correlates with cognitive test scores. Physical interaction with tau is unclear.	(54)
R242-L243	243–441 (Tau-CTF24)	Calpain-1	Accelerates intracellular propagation of tau and has reduced capacity for promoting microtubule assembly compared to tau441.	(55)

*The summary recaps tau fragments, proteases responsible for their generation and the impact on disease pathogenesis in animal models and human kindred*.

*Cas, caspase; CGCs, cerebellar granule cells; NMDAR, N-methyl-D-aspartate receptor (also known as the NMDA receptor); PHFs, paired helical filaments; ADAM10, disintegrin and metalloproteinase domain-containing protein 10, a membrane-tethered protease*.

Several members of the caspase family of proteases have been identified to cleave tau (caspases-1,−3,−6,−7, and−8) at residue 421 (49). This truncated fragment has the propensity to assemble into aggregates faster than the full-length protein and can be detected in fibrillar pathologies of the brain of AD patients (49). The 34 kDa fragment generated by caspase-2, known as ΔTau314, is another well-studied caspase-cleaved tau fragment (51, 78). This C-terminally truncated fragment of tau mis-localizes to dendritic spines and causes cognitive dysfunction in an animal model of tauopathy (51). A recent report indicates elevated levels of this fragment in cognitively impaired human kindreds (78). A 35 kDa N-terminally truncated tau fragment entailing the microtubule-binding repeat domains was reported to be present in post-mortem brains of patients diagnosed with tauopathies in which 4R isoforms predominate (79). Minimal expression of this fragment in mice (Tau35) led to tau neuropathology, deficits in cognitive and motor function, muscle degeneration and impaired proteostasis (51, 80). Since truncation of tau could facilitate subsequent conformational changes and enhance aggregation, modulating this particular PTM in different tauopathies could offer a new approach to therapeutic intervention (80, 81).

### Acetylation

The very first report on tau acetylation was from a study on synthetic peptides spanning amino acids 160–182 and 264–287 of the full-length (2N4R) tau, to generate acetylated-tau antibodies. As a result of this study, Sirtuin 1 (SIRT1) was identified as a deacetylase targeting tau (82). Partially akin to phosphorylation, acetylation has a regulatory role on tau-microtubule interactions (83). There are over 20 Lys residues that can be targeted for acetylation, and acetylation of some appears to be of particular pathological significance (Figure 2C) (82, 84, 85). Tau protein can also undergo autoacetylation, by the help of catalytic Cys residues in the microtubule binding region (86). By neutralizing the repulsion of positively-charged Lys residues, acetylation tends to make parallel stacking of β-strands more favorable and hence promote tau aggregation (84, 87, 88). Physiological investigations have revealed that acetylation of tau also affects degradation of the protein (by inhibiting ubiquitination of Lys residues) and hence slows the rate of protein turnover; this is associated with attenuation of tau microtubule binding and promotion of aggregation, especially into soluble oligomers (89, 90).

### O-glycosylation

O-glycosylation (or O-GlcNAcylation) is a dynamic process that involves the addition of the β-d-N-acetylglucosamine (GlcNAc) molecule to Ser or Thr residues of the target protein via O-linkage. The two enzymes responsible for regulation of this PTM are O-GlcNAc transferase and O-GlcNAcase (91). It has been shown that O-GlcNAcylation can negatively regulate tau phosphorylation in a site-specific manner *in vitro* and *in vivo* (in cell models) (92). The balance between tau hyperphosphorylation and O-glycosylation could also impact the protein's cellular localization (93). Moreover, tau tangles isolated from AD patients are hyperphosphorylated and hypo-O-glycosylated (92). However, it is still not fully clear whether decreased O-GlcNAcylation of tau has any causative effect on hyperphosphorylation or is simply a secondary effect (94). In fact, increasing tau O-GlcNAcylation via inhibition of O-GlcNAcase in JNPL3 tauopathy mouse model [mutant tau P301L under the mouse *PRNP* promoter (95)] hindered tau aggregation and decreased neuronal cell loss by impairing tau's ability to oligomerize and without affecting tau phosphorylation (96).

### Other PTM's

Beyond phosphorylation, proteolysis, acetylation and O-glycosylation, multiple Lys residues occurring in tau molecule (44 residues in human full-length tau variant 2N4R) may be modified by other PTMs (ubiquitination, sumoylation, and methylation), which can then play a role in tau assembly and toxicity via participation in electrostatic and hydrophobic interactions (87, 97). N-glycosylation, prolyl-isomerization, nitration, polyamination, and oxidation are yet other PTMs discussed in relation to the tau-mediated pathogenesis of AD. Even though each tau PTM is formed by a distinct mechanism utilizing different enzymes, cofactors and chemical groups, their net effect is to impact the protein's function, its cellular localization, and turnover (98, 99).

In terms of taking these concepts further, a particular case in point relates to accumulating evidence that sites of tau cleavage may be specific to individual or subgroups of tauopathies, it then being likely that tau fragmentation events may impact the evolution of collections (ensembles) of co-existing alternative tau conformational species (51). This consideration opens a window on the more general issue of non-covalent forms of variation in tau that may impact physiological and pathophysiological output measures.

## Assembly States and Conformations of Tau

### Prion Effects, Conformers, and Templated Misfolding

Although the prion concept was formulated to refer to a specific group of proteinopathies caused by misfolding of the cellular prion protein (PrP^C^), other proteins were subsequently discovered to undergo a similar process. Unlike PrP^C^ which is generated in the secretory pathway, these other proteins such as URE2 and Sup35 in yeast, are located in the cytoplasm. Today, the prion paradigm - according to which a fundamental cause of specific disorders is the misfolding and seeded aggregation of specific proteins—is a useful unifying principle to explore the different facets of pathogenesis of many age-related neurodegenerative diseases. In prion diseases, the processes of protein replication (accumulation of misfolded copies), toxicity and infectivity can be uncoupled in different experimental paradigms, removing the constraint for extending insights from prion disease to non-transmissible neurologic syndromes. Tauopathies came to be considered in this broader context following pioneering works starting in 2009 (100–102). Proteins with this behavior [i.e., tau, alpha-synuclein (α-syn)] have been referred to as “prion-like” or “prions” (103–105). This nomenclature has also been applied to amyloid beta (Abeta) (106), this AD pathogenesis-associated peptide deriving from sequential endoproteolysis of a type I transmembrane protein and secreted into the extracellular space.

**Figure 3:**
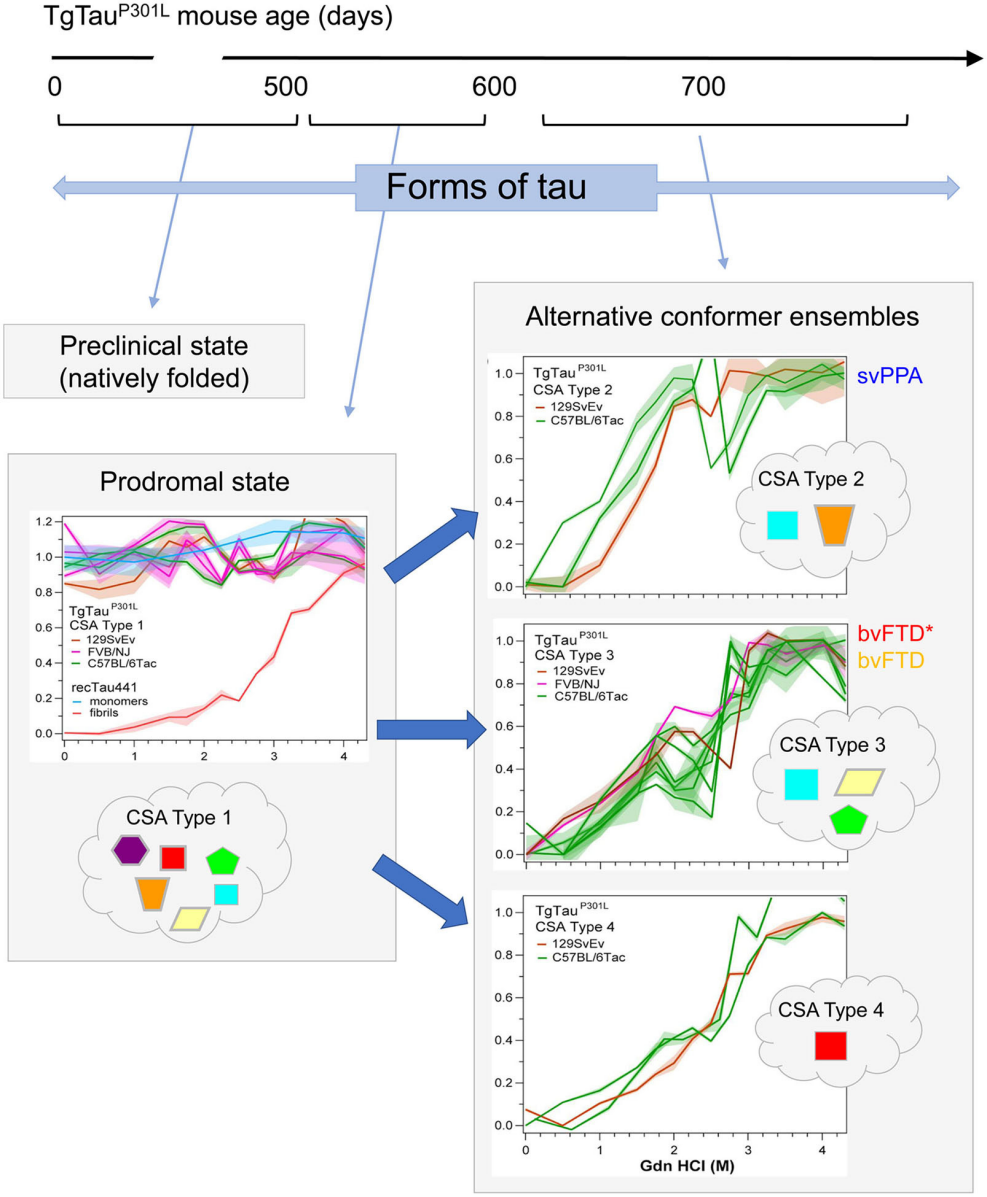
Temporal evolution of conformer ensembles in the pathogenesis of a primary tauopathy. Conformers of protease-sensitive detergent-insoluble tau in TgTau^P301L^ mice are represented by different geometric shapes [modified from (107)]. Different coexisting combinations (i.e., ensembles) of conformers corresponding to different CSA profiles are shown within the cloud outlines while the corresponding CSA traces of the samples are shown above these, with the y-axis representing F_app_ values and the x-axis representing increasing Gdn HCl concentrations up to 4.5 M. Distinct CSA Types 2, 3, and 4 are present in the TgTau^P301L^ mice; each curve represents dissociation and unfolding in one individual. F_app_ values are plotted as mean ± SEM (shades) for each denaturant concentration and assayed in triplicate. Curve analysis was performed with non-linear regression and significance determined with generalized Wilcoxon test. Average ages (days ± *SD*) for CSA Types 1, 2, 3, and 4 were 535 ± 32, 649 ± 56, 629 ± 57, and 682 ± 82 days, respectively and for the types of fibrillar assemblies associated with the CSA profiles, see (107). CSA Types 2–4 are seen in mice with statistically indistinguishable average ages and hence likely represent alternative pathways of ensemble evolution (blue arrows). The closest equivalent human disease profiles to mouse CSA Types 2 and 3 are presented to the right of the CSA plots in the boxes with solid outlines; the initial clinical diagnoses assigned to these FTLD-MAPT-P301L cases are shown (svPPA, semantic variant of primary progressive aphasia; bvFTD, behavioral variant of FTD; bvFTD*, a bvFTD sub-variety). CSA indicates conformational stability assay; F_app_, indicates values of apparent fractional change.

As per the covalently heterogeneous forms of tau listed above, accumulating evidence supports presence of multiple conformationally distinct conformers (strains) of tau (107–111). Acknowledging the existence of widely-used conformation-specific tau antibodies (112) and noting heterogeneity in PrP structure in prion strains (113), a conceptual approach deriving from prion disease is to consider (i) alternative folding outcomes as key determinants of heterogeneity in clinical presentation of tauopathies and (ii) propagation of tau conformers by templating as a mechanism underpinning the spread of disease-associated forms. In our investigations, conformation-dependent immunoassays (CDIs) and conformational stability assays (CSAs) were utilized to appraise abnormally-folded tau. In this procedure tau is first exposed to the protein denaturant guanidine hydrochloride (Gdn HCl) and then exposed to europium-labeled antibody against epitopes that are hidden under native conditions in the absence of Gdn HCl (107, 113). Signal intensities in the absence and presence of Gdn HCl give ratiometric values for how an epitope is hidden in a misfolded molecule (CDI) and stepwise addition of Gdn HCl in a conformational stability assay (CSA) yields a characteristic profile for gradual chemical unfolding—differences in stability profiles have been described for prions and Abeta, providing evidence of strains with distinct conformations (113–115). Most importantly, CDI ratios and CSA unfolding conformational signatures are independent of the concentrations of the misfolded species and the procedure does not involve pre-purification or *in vitro* amplification steps that can alter the *in vivo* conformational repertoire and biological properties of strain isolates (116, 117). For PrP, CSAs differentiate strains regardless of PTMs such as glycosylation and glycolipidation (117–120). For tau we selected a monitoring antibody for epitope occlusion located in the R3/R4 boundary of microtubule binding repeats, an area less decorated by PTMs (121, 122) but also considered to be conformationally remodeled in tau strains (123). The tau CDI assay performed against recombinant full-length human tau (tau441) that was deliberately misfolded into fibrils demonstrated a broad linear range for these assays. Using human FTLD-MAPT-P301L brain material derived from frontal cortex and mouse P301L brain materials we found related, complex unfolding patterns indicative of multiple co-existing conformers (107), leading to a conclusion that the collection (ensemble) of tau conformers seen at disease endpoint evolves from a precursor population, a complex mixture of early misfolded forms (Figure 3).

### Oligomers

While descriptions of cryo-EM data on hallmark of tau fibrillar assemblies present at end-stage are listed below, these assemblies are not necessarily the neurotoxic entities leading to disease and instead oligomers may fulfill this role (as considered in section toxic effects of abnormal tau). Also, soluble, non-fibrillar, oligomers are posited to be responsible for the spread of pathology throughout the brain (124); active seeding capacity may correlate poorly with fibrillar deposits seen by light microscopy and high molecular weight soluble forms of tau derived from size exclusion chromatography may be most adept in *in vitro* seeding reactions (108–110, 125, 126). A rare species of high molecular weight, soluble, phosphorylated tau oligomers present in brain of transgenic tau mice, as well as AD patient cortices are believed to be the endogenous form of tau involved in propagation (127). In accordance with this observation, tau seeding strongly correlates with the amount of oligomeric and phosphorylated tau in post-mortem brains of AD patients, strongly suggesting that oligomeric hyperphosphorylated tau species act as seeds (128). Interestingly, these soluble assemblies demonstrate substantial patient-to-patient heterogeneity, perhaps because they include a larger variety of PTMs in comparison to large, non-soluble fibrils. In turn, these heterogeneities could relate to differences in clinical measures such as rate of clinical decline amongst AD patients (107, 128).

For the foregoing discussions of oligomers, there is a caveat concerning a range of definitions, terminology, and methodologies used for these tau assemblies: dimers (disulfide bond-dependent or -independent), multimers (trimer, tetramer, etc.), granular aggregates or small filamentous protofibrils [defined based on observations made in EM or atomic force microscopy (AFM)] have all been considered within this umbrella term.

### Liquid-Liquid Phase Separation (LLPS) of Tau

Disruptions of membraneless organelles (MLOs) can induce neurodegenerative processes (129–133). MLOs, unlike canonical membrane-bound cellular organelles such as secretory vesicles, the endoplasmic reticulum and mitochondria, do not have an enclosing membrane yet compartmentalize like oil droplets in water (134). Intrinsically disordered proteins containing low complexity domains and RNA molecules can bind to each other and form liquid droplets, a phenomenon known as LLPS that has been known to regulate reversible dynamics of MLOs in cell milieu (134–136).

Alterations in the biophysical properties of MLOs became evident in the context of amyotrophic lateral sclerosis (ALS)/FTD. Pathogenic mutations in TDP-43, hnRNPA1 and FUS perturb disassembly of MLOs (e.g., stress granules) and predispose to aggregate into amyloid-like fibrils (130–133). Similarly, toxic dipeptide repeat proteins produced from hexanucleotide repeat expansion in chromosome 9 open reading frame 72 (C9ORF72) bind to low sequence complexity domains in RNA-binding proteins; these binding events subsequently interfere with physiological functions carried out by multiple MLOs and in this way contribute to pathogenesis (129). More recently, several lines of evidence suggest that intrinsically disordered structure, inhomogeneous charge distribution, hyperphosphorylation, and/or aggregation-prone mutations allow tau to undergo LLPS under conditions of molecular crowding (137–143). While the *in vivo* parameters and co-factors involved in the LLPS of tau are not fully understood, sustained conditions can coerce droplets to more solid-like forms; for example, conversion to irreversible hydrogels and amyloid-like fibrils in the presence of multivalent polymers (e.g., RNAs) or pathogenic mutations (137, 142). Presumably, the liquid-solid phase transitions would, in turn, trigger regulated cell death starting within the preclinical stage of tauopathies in a similar way to ALS/FTD (129, 130). The molecular mechanism underlying LLPS of tau remains challenging to assess *in vivo*, due to the metastable and reversible property of liquid condensates. Nonetheless, these findings indicate that the demixed state of tau droplets can act as a possible toxic intermediator which occurs in a transitional state between internalization and intracellular tau propagation.

### Tau Structures Deduced by Cryo-EM

Recent examples of a variety of atomic-level resolution structures for tau fibrils obtained by cryo-EM examination of brain material (87, 123, 144–146) represent milestones in the field as they provide molecular coordinates for designed ligands and capture in still-life variations in what some might term tau strains. Knowledge at the structural level of tau fibrils before the cryo-EM era was insufficient; although solid-state nuclear magnetic resonance (NMR) and electron paramagnetic resonance (EPR) were able to assign strands to certain peptides in synthetic fibers, no atomic model was available (147). Cryo-EM studies of tau fibrils obtained from brain of human patients with distinct tauopathies [AD, PiD and chronic traumatic encephalopathy (CTE)] have revealed that each tauopathy has characteristic filament folds, which are conserved among individuals with the same disease, yet different from structures obtained from *in vitro* aggregation of recombinant tau (148–150). The first report on cryo-EM structure of pathological tau (with 3.4-3.5 Å resolution) is based on atomic models of PHFs and straight filaments (SFs) obtained from an individual AD patient. This structure shows that the core of both tau filaments is made of identical protofilaments (residues Val306-Phe378) which adopt a combined cross-β/β-helix structure, and the two types of filaments are ultrastructural polymorphs with differences in their inter-protofilament packing (145). The ultrastructure of tau filaments obtained from PiD and CTE came along next (with resolution of 3.2 Å and 2.3 Å, respectively) (144, 146). While the filament core in PiD (a 3R tauopathy) consists of residues Lys254–Phe378 of 3R tau, the filaments in CTE entail residues Lys274–Arg379 of 3R and Ser305–Arg379 of 4R tau isoforms (144, 146). Nonetheless, this current repertoire of folds is superficially narrower than for other types of analyses. Besides technical considerations relating to sampling, there may be intrinsic reasons for this disparity. It could be that soluble tau oligomers exist in multiple conformations, but only a subset of these conformations is represented by the structures present in long-lived fibrils. Alternatively, despite a few common ultrastructures, PTM patterns could add another level of conformational diversity (107, 128). As an example, ubiquitination of tau within the fibril forming core region (Lys369–Glu380) can mediate fibril diversity (87).

## Cell Lineages Harboring Abnormal Forms of Tau

Analyses of cell-free systems or purified protein from autopsy material cannot encompass dynamic relationships applying to genesis and turnover of tau conformers in living cells, nor to the important situation in the sub-clinical phase of disease where therapeutic interventions might best be applied before irrevocable neuronal loss. In prion disease, strains made of different conformers of the pathogenic infectious prion protein (PrP^Sc^) are often considered to have differing abilities to infect cells; this effect has been studied by using endpoint-titrated samples obtained by serial dilution (i.e., biologically cloned) to infect susceptible animals, which are then in turn scored for different neuropathological patterns of protein accumulation (151). In a seemingly parallel set of observations to protein structural assays, different tauopathies are known to be associated with different cell populations. Thus, (i) 3R tauopathies include PiD with 3R tau in neuronal cytoplasmic inclusions called Pick Bodies (11); (ii) 4R tauopathies such as CBD include glial cells of the cortex and white matter as well as neuronal accumulation; similarly, in the case of PSP, there are tau tangles in glia and neurons (152). Both CBD and PSP may also include oligodendroglial tau inclusions (11); (iii) 3R**+**4R tauopathies also exist and are most commonly represented by AD, with tau in neuronal cell bodies as NFTs and within dystrophic neurites lying nearby mature plaques. Noting these points and, because it is known that experimentally-tractable FTLD-MAPT tauopathy manifests in different cell populations sharing the same *MAPT* genotype (108, 153, 154), it is likely that aspects of the cell biology of tau remain to be discovered. It has been reported that synthetic tau-preformed fibrils and pathological tau derived from brains of AD patient are capable of causing tau aggregation in both cultured cells and wild type mice (155, 156), and that the cell-to-cell spread pattern of the seed-competent tau conformers in the central nervous system (CNS) was determined by synaptic connectivity (e.g., afferent and efferent connections) rather than spatial proximity (156–159). Moreover, similar to prion strains, tau conformers derived from distinct tauopathies including AD, PSP, and CBD recapitulated their phenotype characteristics of tau pathology; time-, dose-, and injection site-dependent patterns of spreading and cell type-specific aggregation (159, 160) [also reviewed in (161)]. In prion diseases, a popular idea is that different prion strains perpetuated by experimental inoculation prefer to infect different cells (a concept commonly called tropism) (151) but primary tauopathies derive from germline mutations and contributions of exogenous infection to this process may not exist or may be secondary events. Nonetheless, for malformed tau emerging spontaneously within the CNS, uptake by different cell lineages could play an active role in selective propagation of tau strains, this arising as a consequence of fundamental differences in endogenous processes that distinguish neurons, oligodendrocytes and astrocytes. This latter concept might begin to explain why conformer mixtures can often be encountered within the same brain (107).

### Tissue Tropism of Tau Conformers (Glial Tauopathies)

Tau expression is predominantly present in neurons, with lower expression levels or signals below assay threshold applying to oligodendrocytes and astrocytes (160, 162). In the secondary tauopathy AD, tau aggregates are only found in neurons as NFTs and neuropil threads, which are composed of both 3R and 4R tau (163). On the other hand, abundant glial tau deposits are found along with neuronal pathology in primary tauopathies and in other subtypes of FTD/FTLD including PiD, CBD, PSP, GGT, and AGD (1). The majority of glial tau pathologies are observed in oligodendrocytes and astrocytes and, in some instances, tau inclusions are also found in microglia (164, 165). Interestingly, in a neuronal tau knockdown mouse model (TauKDn^cre;fl/fl^), oligodendrocytic tauopathy spread through adjacent brain regions, whereas astrocytic inclusions remained confined to the injection site (160). The various deposition morphologies of glial tau (1, 166) (described below) may have functional correlates and could be drivers underlying the diverse manifestations of neurodegenerative tauopathies.

***Ramified inclusions*** are astrocytic tau fibrils found in PiD. Thick processes and eccentric nuclei are accompanied with ramified inclusions (167, 168).

***Tufted tau inclusions*** are densely packed fibrils found in the proximal processes surrounding astrocytic nuclei and are the pathological signature of PSP. Morphologically, star-like tufts of dense fibers emanate from the cell body (169, 170).

***Coiled bodies*** are intracytoplasmic tau inclusions surrounding the nucleus of oligodendrocytes that form coil-like or comma-like inclusions. They are also common in many FTLD-subtypes such as PSP, CBD, AGD, and FTLD-MAPT-P301L (169, 171, 172).

***Astrocytic plaques*** are hallmarks of CBD and take the forms of densely tau-immunoreactive stubby dilatations in distal processes of astrocytes (173). The inclusions are comprised of twisted and straight tubules with diameters of 15–20 nm (174).

***Argyrophilic threads*** are tau-positive thread-like structures in the processes of astrocytes and oligodendrocytes and are prominent in CBD (11, 175).

***Thorn-shaped inclusions*** are juxtanuclear assemblies with tau-immunoreactivity and extension into the proximal processes of astrocytic endfeet at the pial surface and around blood vessels (173). These appear as argyrophilic masses with flame or thorn-like shapes in both PSP and aging-related tau astrogliopathy (ARTAG) (173, 176).

***Globular oligodendrocytic and astrocytic inclusions*** comprise insoluble globules and granular tau deposits emanating from the cell body that are unique characteristics of GGT (11, 177, 178).

***Pick-bodies*** are neuronal tau inclusions found in PiD and are round in shape but to a lesser extent, ***Pick body-like inclusions*** are also evident in both astrocytes and oligodendroglia in PiD (167, 168).

Different manifestations of astroglial tau are recently reviewed and summarized by Kovacs (166), along with a consideration of potential precursor forms. These data point to a non-trivial role for astroglial tau in pathogenesis of diverse diseases. On the other hand, there seems to be a molecular conundrum regarding the origins of the tau conformers fueling these diverse glial tau pathologies, noting that glial expression of tau mRNA in human tissue is much lower than in neurons and (16, 18) that tau transgenic mice using the *PRNP* promoter (generally considered to drive pan-neuronal expression) nonetheless accumulate hyperphosphorylated and argyrophilic tau in astrocytes (154, 179).

Glial cells constitute roughly half of the cells of the human CNS (180). In healthy conditions, they considerably influence nervous system development, from neuronal birth, migration, axon specification, and growth through circuit assembly and synaptogenesis (181), while in CNS injury, they are responsible for phagocytosis and elimination of microbes, dead cells, and protein aggregates, as well as other particulate and soluble antigens that may endanger the CNS (182, 183). The glial pathologies could be contributed by a cell-to-cell transfer initiated by exocytosis, budding from plasma membrane and synaptic secretion of cellular and pathogenic tau to the extracellular space, these mechanisms having long been considered as common mechanisms for disease progression in most neurodegenerative diseases (184). A simple extrapolation is that glial tau inclusions are derived from a neuronal source by the active cellular process of efferocytosis; this is a defense mechanism during the resolution of pathological events that involves engulfment and clearance of dead and dying cells by the professional phagocyte (e.g., microglia) and non-professional phagocytes (e.g., oligodendrocytes, astrocytes, neuronal progenitor cells) in the CNS (185). This simple idea starts to address the conundrum presented by most tau expression deriving from neurons, but is not a comprehensive explanation; thus the syndrome called ARTAG (166, 173) has astrocytic tau without neuronal tau accumulation and oligodendroglial tau can be detected in young to middle-aged TgTau^P301L^ mice in the apparent absence of neuronal tau inclusions, which may not appear until many months later (108).

## Spread of Tau at the Cellular and Tissue Level

In early stages of most protein misfolding diseases, the pathological changes, including aggregated protein accumulation and neurological dysfunctions are restricted to confined regions of the nervous system. However, as the disease progresses such alterations spread throughout the CNS, suggesting the presence of a cell biological spreading mechanisms for misfolded protein species (186), with these not necessarily being synonymous with conformational templating mechanisms, some of which may take place inside cells. These general thoughts have become embodied in the specific idea that neuropathological staging of tauopathies originally mapped with phospho-specific antibodies and conformation-dependent antibodies (187–190) reflects the sequential spread of misfolded tau species, following patterns of neuroanatomical connectivity rather than simple physical or spatial proximity (191–193).

### Generalized Transfer Processes Between Homologous Cells

**Figure 4:**
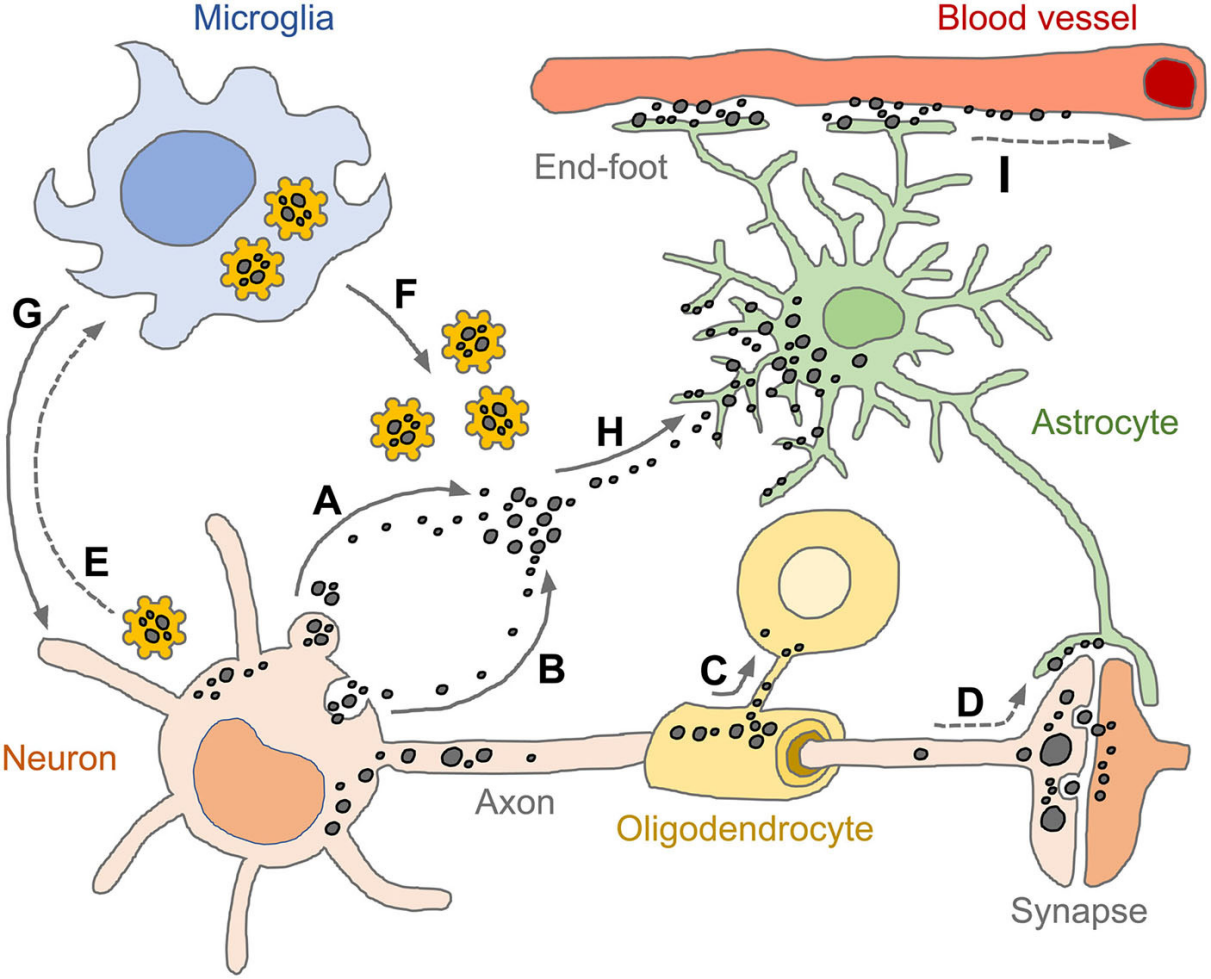
Spread of pathogenic tau conformers and glial tauopathy. Under disease conditions, tau is dissociated from axonal microtubule structure, aggregated (or condensed) and relocalized in somato-dendritic compartments. The pathogenic tau conformers enhance cytoplasmic proteostasis pathways and promote secretion machinery, such as ectosomes **(A)** and exosomes **(B)**, which aim to get rid of the cellular burdens. Oligodendrocytes transmit tau pathology through their own processes near the formation of the myelin sheath **(C)**. Tau conformers also spread to neighboring neurons and astrocytes *via* post-synaptic clefts and astrocytic processes, respectively **(D)**. Intracellular tau aggregates are sensed by pattern recognition receptors (e.g., NLRP3) and primed microglia **(E)**. The reactive microglia amplify innate immune responses by releasing pro-inflammatory effector molecules (e.g., tau-NLRP3-inflammasomes, cogwheel shapes in yellow) **(F)** and subsequently induce pyroptosis which is an inflammatory form of regulated cell death **(G)**. Extracellular tau conformers transferred to astrocytes through both ingestion and phagocytosis **(H)**, appearing as diverse tau pathologies. Tau immunoreactivities are also found in perivascular astrocyte end-feet, indicating that astrocytic tau inclusions could be drained through glymphatic system which is a fluid-clearance pathway in the brain tissue **(I)**. Tau conformers are shown as black dots in various sizes. Solid line arrows indicate experimentally proven pathways (published), while dotted line arrows indicate pathways as yet unidentified.

Intracellular depositions of abnormally folded proteins act as dangerous molecular signals (DAMPs, damage associated molecular patterns) causing stress conditions and provoking diverse responses which can address burdens such as accumulation of misfolded tau, α-syn, Abeta, TDP-43, and PrP^Sc^ by upregulating proteolysis and/or secretion pathways (184). Depending on the secretion pathways (e.g., membrane fusion, ectosomes and exosomes), secreted tau can be found as a free protein and/or within vesicles. However, secretion is not the end of the story and nor is it necessarily a good outcome for the tissue; once bound to the plasma membrane of neighboring cells, tau conformers may yet be internalized by endocytosis, pinocytosis or phagocytosis (regardless of the type of the adjacent cell) (Figure 4) (184, 194). To complete this process, extracellular tau conformers may be required to interact with phosphatidylinositol 4,5 phosphate (PI(4,5)P2), cholesterol, sphingolipids and/or heparan sulfate proteoglycans located at the extracellular leaflet of the plasma membrane (195–197). Secreted tau in a vesicular form (e.g., ectosomes and exosomes) can fuse to the plasma membrane or get endocytosed by recipient cells. Exosomes are released on the exocytosis of multivesicular bodies following inward budding of the outer endosomal membrane. Ectosomes are formed by outward budding of the plasma membrane and can deliver larger cargos (194, 198). Heparan sulfate proteoglycan-mediated macropinocytosis is another type of endocytosis that is the preferred entry for tau monomers and oligomers (197, 199). Pathogenic tau conformers can also travel directly between cells via tunneling nanotubes, these being actin-rich membranous protrusions that allow for intercellular transport of various cargos not only between neurons but also astrocytes (200). However, it remains unclear how the internalized tau conformers in recipient cells escape from endosomal (or lysosomal) processing and in turn encounter endogenous substrates for templated fibrillization (161).

### Heterologous Transfers

In terms of different lineage origins for donor and recipient cells, there is an emerging stream of literature from seeding paradigms illustrating different pairwise combinations. Indeed, one might imagine six permutations of *heterologous* one-way transfer of abnormal tau between neurons, astrocytes and oligodendrocytes. While the issue of *MAPT* gene expression crops up again here, i.e., the ability of all three lineages in the human brain or transgenic mouse models to express different spliced tau mRNAs (given native tau substrate is required for propagation by templated misfolding), some permutations are already established. In one experimental configuration, the source of malformed tau can derive from a clinical syndrome with multiple affected lineages, hence a heterogeneous tau source, which then allows for the detection of responses in different recipient lineages. Using source material from tau transgenic mice with extensive pathology or from human disease tissue, induced tau pathologies after seeding into indicator mice are not restricted to neurons but also include astrocytic and oligodendrocytic inclusions. Seeding experiments using stereotaxic injections into different neuroanatomical areas (for example, into the corpus callosum, to examine oligodendrocyte responses) allow insight into lineage tropism effects, the contribution of neuroanatomical pathways and trans-synaptic spread and comparisons with staging schemes derived solely from examination of human brain material (100, 158, 159, 169, 187, 188, 201–203).

### Tissue Level Effects; Role of the Glymphatic System

Emerging evidence suggests the existence of a mechanism underlying solute clearance from the brain's extracellular space, this being termed the glymphatic pathway. Unlike traditional degradation processes including autophagy and UPS, this pathway conveys protein aggregates from the parenchyma to the CSF as a highly organized fluid transport and clearance system (204–206). This pathway facilitates the flow of CSF to arterial perivascular space and subsequently into the brain interstitium which contains pathogenic tau conformers released from neurons and glia. The flow then migrates toward the venous perivascular spaces, clearing solutes from the neuropil into meningeal and cervical lymphatic drainage vessels. The astrocytic aquaporin-4 (AQP4) water channels localized in astrocytic end feet play an important role in CSF-ISF exchanges in both periarterial and perivenous spaces (207). Animals lacking AQP4 gene expression exhibit a ~70% reduction in interstitial solute clearance compared to wild-type control mice (205). Depletion of AQP4 also exacerbated neuropathology by increasing levels of phosphorylated tau and reactive gliosis in a mouse model of traumatic brain injury (TBI) (204). Pharmacological inhibition of AQP4 using TGN-020 (N-1,3,4-thiadiazol-2-yl-3-pyridinecarboxamide) impaired glymphatic CSF-ISF exchange and tau protein clearance in rTg4510 tau transgenic mouse model (208). Given that thorn-shaped tau inclusions at astrocytic end-feet are hallmarks in both PSP and ARTAG (166, 173), these data may suggest an intriguing connection (or competition) between pathologic spread of toxic tau conformers vs. inactivation of proteinaceous pathological tau seeds (Figure 4) (184, 207). Further studies are needed to substantiate the dual and opposing roles of glial cells in tauopathies, being both beneficial and detrimental.

## Toxic Effects of Abnormal Tau

Although results obtained from numerous studies indicate that misfolded protein aggregates are toxic to neurons *in vitro* and *in vivo*, the molecular mechanism(s) through which they induce their toxicity is not always well-established. This is partially due to the heterogeneity of aggregated and misfolded proteins species. Since misfolded proteins can co-exist in several distinct forms with different features and characteristics, they might each induce neurotoxicity in their own idiosyncratic ways. These general considerations about neurodegenerative disease very much apply to tau, a protein with impressive diversity of covalent forms and conformers and an ability to assemble into supramolecular structures in neuronal, astrocytic and oligodendroglial lineages.

It is known that the neuronal loss in AD patients exceed the number of NFTs (89), and neurons containing NFTs are functionally intact *in vivo* (209, 210). Moreover, some studies in animal models have shown that overexpression of tau can lead to cell death and synaptic dysfunction in the absence of tau filaments (211). In fact, reducing tau overexpression in mutant tau transgenic mice (rTg4510) decreases neuronal cell loss despite progressive formation of tau tangles (212). At the same time, the onset of clinical symptoms in AD and PSP brains correlate with elevated levels of multimeric, soluble assemblies, known as tau oligomers. Empirically, one way to test for toxicity is by direct injection of purified material and here it is noted that injection of oligomers into the brain of wild-type mice, rather than monomers or fibrils cause cognitive, synaptic, and mitochondrial abnormalities (126, 213). Collectively, these points suggest that formation of tau tangles (or tangle-like structures) is not essential for neuronal loss and that tau-induced neurotoxicity is in fact dependent upon the formation of non-filamentous, aggregate intermediates known as tau oligomers (89, 214).

### Inflammation and Gliosis

Since brain tissue is immune-privileged with the restricted access of immune cells through blood-brain barrier, resident microglia, monocyte and astrocytes are the major effector cells of the innate immune defense against microbial infection, brain injury and neurodegenerative disorders (215). Neuroinflammation in various proteinopathies, where protein aggregates are causing cell damage, is induced by CNS-resident and/or potentially blood-derived innate immune cells. On the other hand, adaptive immune cells such as B and T lymphocytes drive the pathological processes (216) in microbial infections and autoimmune disease (e.g., encephalitides and multiple sclerosis, MS).

The components of the innate immune system have their own inherent protective and defensive functions against various danger signals (DAMPs) as well as pathogens (pathogen-associated molecular patterns, PAMPs), while excessive or non-resolving immune responses have the opposite effect and may damage the host (217, 218). Pathogenic protein conformers in various neurodegenerative diseases have been reported to activate chronic neuroinflammation through pattern recognition receptors which are important sensors of innate immunity found in most CNS cells. For example, oligomeric forms of Abeta and α-syn induce NF-κB-dependent pro-inflammatory gene expression by binding to cell surface receptors such as receptor for advanced glycation end products (RAGE), toll-like receptor (TLR)-2 and TLR-4 (219–222). Extracellular soluble forms of Abeta are internalized into microglia by binding to a protein, triggering receptor expressed on myeloid cells 2 (TREM2), which is capable of promoting phagocytic activity via regulation of C/EBPα and CD36 expression (223). It is known that aggregation-prone proteins including Abeta, α-syn, TDP-43, and superoxide dismutase 1 (SOD1) along with other DAMPs, such as ATP and lysophosphatidylcholine can activate intracellular inflammasomes following interleukin 1 beta (IL-1β) release (224–229).

Concerning tauopathies, there is increasing evidence that inflammasome-mediated gliosis and innate immune responses are recurrent features (230, 231). One may speculate that pathogenic tau conformers taken up into glial cells could act as endogenous DAMPs and be recognized by cytoplasmic pattern recognition receptors such as inflammasomes (232, 233), molecular assemblies which are expressed and activated in different types of CNS-resident cells (231, 234). There is a critical role for the inflammasome-mediated innate immune responses in tau pathogenesis, given that exogenously and non-exogenously seeded tau could activate inflammasomes (232, 233). Upon activation, inflammasome components referred to as PYD and CARD form protein filaments. These polymerization steps are conserved signaling cascades in innate immunity and inflammation (233, 235) and are somewhat “prion-like” as assembly of the ASC specks can transfer to neighboring cells (Figure 4) (236, 237). In transgenic mice expressing human MAPT-P301S tau (MAPT^P301S^PS19), the ablation of senescent astrocytes and microglia prevents gliosis, deposition of tangle-like structures, degeneration of cortical and hippocampal neurons, indicating the role of dysregulated glial cells that could initiate and exacerbate tau pathology (238).

### Disruption of Cellular/Axonal Transport

Growing evidence suggests that defective neuronal and axonal transport due to early axonal dysfunction could play a contributory role in several neurodegenerative diseases. Standing somewhat in contrast to the lack of deficits in tau knock-out mice (26, 27, 239, 240), there are reports to this effect for tauopathies at their early disease stages (214, 241, 242). In fact, several studies have demonstrated that the most common tauopathies are characterized by several features that point to a significant role for axonal dysfunction that may originate from deficits in fast axonal transport (243–246). One report has proposed that tau oligomers disrupt microtubule stability and trafficking, thus affecting organelle distribution, and inducing toxicity (247). Oligomers can also cause dramatic displacement of endogenous axonal tau into the somato-dendritic compartments, and, in turn dysregulation of microtubule-based fast axonal transport (248).

### Disruption of Nuclear Cytoplasmic Transport

Declines in the structural integrity of nuclear pore complex (NPC) and the efficiency of nuclear-cytoplasmic transport (NCT) have been reported in neurodegenerative disorders including FTD, ALS, Huntington's disease and tauopathies (71, 74, 249–254). The constant flow of protein and RNA species is critical for transcriptional regulation, signal transduction, cell growth, and cell cycle (255, 256); these molecular transportation events occur through the NPCs, which are one of the largest embedded macromolecular assemblies of the nuclear envelope and form a channel by fusing the outer and inner nuclear envelope leaflets (256–258). NCT through these pores is mediated by around 30 different nucleoporins (NUPs), which are protein building blocks of NPCs and have remarkably long-lifespans (259).

Nuclear localization of tau species and their interaction with DNA have suggested a protective role in genome surveillance for normal cells. Conversely, in disease conditions such as AD, an alteration of these functions might enhance genomic vulnerability and neurodegeneration (32). More recently, impairment of NCT has been reported in transgenic mice expressing P301L tau and in AD brains, wherein mislocalization of NUPs is observed with aggregated tau. Concomitant decreases in the levels of NUPs, especially NUPs rich in phenylalanine-glycine repeats (e.g., NUP-98), suggest deterioration of NPC function (71). Others have shown that pathogenic mutations in *MAPT* caused mislocalization of tau into the somato-dendritic compartment and deformation of nuclear membrane as appraised by lamin B staining of nuclear lamina, consequently interfering with NCT (74). P301S and P301L mutant forms of tau may induce mitotic spindle defects during cell division and produce aneuploid cells prone to apoptosis, with these inferences being supported by analyses of brain cell suspensions derived from corresponding transgenic mice (260). Since the aneuploidy-mediated regulated cell death requires cell division, this type of pathogenic event may be more relevant to glial tau pathologies.

## Discussion

An emerging area of consensus is the remarkable level of diversity of tau, with implications for the lab, for the clinic and for pharmaceutical companies. In the human context—as briefly inventoried here—there are different tauopathies, but as noted above, heterogeneity can also be evident within a given disease entity having the exact same *MAPT* protein coding sequence, as noted above and recently illustrated for FTLD-MAPT-P301L cases (107). An analogous effect is now documented for AD with wild-type human tau isoforms (128). This effect/challenge being accepted, therapeutic approaches using small molecule compounds might nonetheless need to pass the checkpoint of validation in animal models. Perhaps surprisingly, close inspection of mouse models of tauopathy can reveal heterogeneity too.

One general way to explain heterogeneity in biological systems is via the action of modifier genes. Allelic forms of the apolipoprotein E (ApoE) gene are potent modifiers for both genetic and sporadic forms of AD, but in the context of FTLD, citations for their impact are sparse. Also, for use of animal models of tauopathy, there are no high frequency polymorphisms in mice equivalent to the human ApoE e2, e3, and e4 forms affecting residues 112 and 158. While a mouse variant in residue 163 has been described that may originate from the DBA/2 background (261), in our own studies phenotypic heterogeneity was observed in TgTau^P301L^ mice inbred to three backgrounds other than DBA/2 (108). For these transgenic mice, we considered whether a somatic mutation of the MAPT-P301L transgene might offer an explanation for heterogeneity in the CNS phenotypes but a PCR assay for genome rearrangements (262) failed to yield evidence for re-integrated transgene copies in brain genomic DNA—this assay had a detection limit for altered transgenes 1,300x below the level of an endogenous single-copy gene (107). We concluded that variations in the nuclear genome are unlikely causes of disease heterogeneity. While another type of genome, the microbiome, might ultimately have a bearing upon phenotypic heterogeneity, its association with FTD and FTLD has been less explored than in the context of Parkinson's disease (PD) and accumulation of α-syn (263, 264). One might then conclude that heterogeneity of tau species observed in the lab recapitulates an intrinsic biological effect and not a distortion arising in the course of animal modeling.

A widespread assumption when using models has been that animals of the same age and genotype are phenotypically identical; indeed, one might not embark upon testing a therapy in a model if not subscribing to this unwritten assumption. However, while this view may have originated from an earlier era with extensive use of over-expresser transgenic mouse lines with a compressed timescale for pathogenesis and hence lower husbandry costs, it may be inadequate and need reconsideration (265, 266). Instead, deviations from homogeneity in slow pathogenesis models might be telling us that processes are nuanced enough in these animals to capture the very same biological mechanisms that are driving heterogeneity in human tauopathies. In terms of the molecular mechanisms driving heterogeneity, there is no shortage of possibilities. As inventoried in the section on tau physiology, spliced forms and posttranslational covalent variations, there is a thicket of PTMs for tau (phosphorylation, acetylation, O-glycosylation, ubiquitination, etc.), quite beside the protein having six different primary structures due to alternative RNA splicing. Additional layers of complexity might be imparted as tau transits between cell lineages and neuroanatomical areas, across synapses, across areas of the extracellular matrix with different surveilling cells (Sections cell lineages harboring abnormal forms of tau, spread of tau at the cellular and tissue level, and toxic effects of abnormal tau), all or any of which might impose different spectra of PTM enzymes and proteostatic environments. Nonetheless, as tauopathies (a) can be devastating and are a considerable burden on the healthcare system and (b) can occur in the context of comorbidities, means must be sought to stratify these variations to deal with the most important entities. In practical terms, the complex landscape of tau biology can be approached by placing a focus on a foreground species, e.g., ones that are thought to be particularly toxic. Thus, although heterogeneity in the here and now is an “inconvenient truth,” embracing this effect, defining its origins and then adjusting approaches may pave the way for more sophisticated testing and more realistic interventions.

## Author Contributions

S-GK: conceptualization, validation, investigation, visualization, writing—original draft, writing—review, and editing. GE-S: investigation, visualization, writing—original draft, writing—review, and editing. LH: data curation, investigation, writing—review, and editing. JS: conceptualization, supervision, funding acquisition, writing—review, and editing. DW: conceptualization, supervision, funding acquisition, validation, project administration, writing—original draft, writing—review, and editing. All authors contributed to the article and approved the submitted version.

## Conflict of Interest

The authors declare that the research was conducted in the absence of any commercial or financial relationships that could be construed as a potential conflict of interest.
